# MetaDegron: multimodal feature-integrated protein language model for predicting E3 ligase targeted degrons

**DOI:** 10.1093/bib/bbae519

**Published:** 2024-10-21

**Authors:** Mengqiu Zheng, Shaofeng Lin, Kunqi Chen, Ruifeng Hu, Liming Wang, Zhongming Zhao, Haodong Xu

**Affiliations:** Department of Orthopaedics, The Second Xiangya Hospital, Central South University, Changsha, Hunan 410011, China; Key Laboratory of Gastrointestinal Cancer (Fujian Medical University), Ministry of Education, School of Basic Medical Sciences, Fuzhou 350004, China; Fujian Key Laboratory of Tumor Microbiology, School of Basic Medical Sciences, Fujian Medical University, Fuzhou 350004, China; Key Laboratory of Gastrointestinal Cancer (Fujian Medical University), Ministry of Education, School of Basic Medical Sciences, Fuzhou 350004, China; Fujian Key Laboratory of Tumor Microbiology, School of Basic Medical Sciences, Fujian Medical University, Fuzhou 350004, China; Center for Precision Health, McWilliams School of Biomedical Informatics, The University of Texas Health Science Center at Houston, Houston, TX 77030, United States; School of Biomedical Science, Hunan University, Changsha, Hunan, China; Center for Precision Health, McWilliams School of Biomedical Informatics, The University of Texas Health Science Center at Houston, Houston, TX 77030, United States; MD Anderson Cancer Center UTHealth Graduate School of Biomedical Sciences, Houston, TX 77030, United States; Department of Orthopaedics, The Second Xiangya Hospital, Central South University, Changsha, Hunan 410011, China; Center for Precision Health, McWilliams School of Biomedical Informatics, The University of Texas Health Science Center at Houston, Houston, TX 77030, United States

**Keywords:** targeted protein degradation, ubiquitin-proteasome system, E3 ligase, degrons, deep-learning, web server

## Abstract

Protein degradation through the ubiquitin proteasome system at the spatial and temporal regulation is essential for many cellular processes. E3 ligases and degradation signals (degrons), the sequences they recognize in the target proteins, are key parts of the ubiquitin-mediated proteolysis, and their interactions determine the degradation specificity and maintain cellular homeostasis. To date, only a limited number of targeted degron instances have been identified, and their properties are not yet fully characterized. To tackle on this challenge, here we develop a novel deep-learning framework, namely MetaDegron, for predicting E3 ligase targeted degron by integrating the protein language model and comprehensive featurization strategies. Through extensive evaluations using benchmark datasets and comparison with existing method, such as Degpred, we demonstrate the superior performance of MetaDegron. Among functional features, MetaDegron allows batch prediction of targeted degrons of 21 E3 ligases, and provides functional annotations and visualization of multiple degron-related structural and physicochemical features. MetaDegron is freely available at http://modinfor.com/MetaDegron/. We anticipate that MetaDegron will serve as a useful tool for the clinical and translational community to elucidate the mechanisms of regulation of protein homeostasis, cancer research, and drug development.

## Introduction

Cells employ protein degradation to eliminate damaged, abnormal, misfolded, and other unnecessary proteins [1, 2]. In eukaryotic cells, protein degradation primarily occurs through the ubiquitin-proteasome system (UPS) [3]. This process is not only vital for maintaining protein homeostasis but also essential for ensuring the proper functioning of cellular processes such as cell cycle progression, signal transduction, differentiation, and growth [4, 5]. Dysfunction in protein degradation can lead to various diseases, including malignant tumors and neurodegenerative disorders [6–8]. Ubiquitin (Ub), a highly conserved protein composed of 76 amino acids with a molecular weight of 8 kDa, plays a central role in this process. The coordinated action of ubiquitin ligases, including the E1: Ub-activating enzyme, E2: Ub-conjugating enzyme, and E3: Ub-ligase, triggers a cascade reaction that attaches ubiquitin to the protein designated for degradation, forming a ubiquitinated protein complex. In the initial step, E1 activates Ub in the presence of adenosine triphosphate and binds the C-terminus of Ub to the active site of E1 (step 1). Subsequently, the activated Ub binds to the cysteine residue in the active site of E2, transferring the activated Ub to E2 (step 2). With the catalytic assistance of E3, the carboxyl-terminal glycine of ubiquitin conjugates to the amino group of the substrate protein residue, typically lysine, resulting in the ubiquitination of the substrate protein (step 3) [9–11].

Degradation signals (degrons) are short linear amino acid motifs, located on target protein substrates [12–14]. When a protein receives a degradation signal, the degron becomes exposed and recognized, after which it is bound by E3 ubiquitin ligase [15, 16]. This binding facilitates the entry of ubiquitinated protein substrates into the enzyme network centered on the 26S proteasome for degradation [17]. The interaction between E3 ligase and the degron is highly specific, playing a pivotal role in determining degradation specificity and maintaining cellular homeostasis [18–20]. An example of this mechanism is the degradation-specific binding of MDM2 (E3) to the degron in the tumor suppressor protein p53, which facilitates the targeted degradation of p53 [14, 21, 22]. Under normal conditions, despite continuous production, p53 is kept at low levels in the cytoplasm due to its ongoing degradation by the UPS. However, when cells are stimulated by external signals, changes in the degron sequence or structure prevents binding to MDM2, resulting in abnormal accumulation of p53 within the cell. This abnormal accumulation then triggers a series of cellular cytotoxic effects, such as cell cycle arrest and apoptosis [6, 23]. The identification of E3-degron interactions is fundamental to understanding the dynamic regulation of proteins.

Recently, the development of high-throughput experimental techniques and proteomics technologies has expanded our understanding of E3s and degrons within the UPS, thereby accelerating the development of targeted protein degradation-based drug therapies and bringing hope to numerous patients [13, 23–29]. However, the identification of degrons remains challenging due to the undetermined substrates for most E3s. The nature of degron and publicly available data sets make it possible to develop computational methods to identify it using pattern recognition and machine learning techniques, which facilitates the development of a number of bioinformatic resources/tools available for degron identification [30–32]. The APC/C degron repository provides valuable insights into the determinants of APC/C degron sequences [33], encompassing information regarding to disordered regions and post-translational modifications. Complementarily, the Eukaryotic Linear Motif (ELM) resource catalogues numerous degron motifs present in proteins [34]. Specifically, DegronMD serves as a novel resource tailored for the comprehensive exploration of degrons, encompassing associated functional aberrations and responses to pharmacological treatments [35]. This resource contains 23 distinct internal degrons within the human proteome. Augmenting these bioinformatic repositories, deep learning models such as Degpred [30] and deepDegron [28] have emerged for the prediction of degrons and the assessment of their perturbations by mutations, respectively. Notably, deepDegron predicts the likelihood of a protein sequence harboring N- or C-terminal degrons, mandating specific mutation input files. Conversely, Degpred leverages a BERT-based architecture to primarily predict internal degrons based solely on sequence information. Additionally, DEGRONOPEDIA [31] has been introduced as a novel web server dedicated to the identification and analysis of degron motifs within proteins, enabling the prediction of potential N-/C-degrons subsequent to proteolytic events (summarized in Supplementary Table S1).

Although the early methods for screening the binding of E3 targeted degrons showed promising, the majority of the models were developed by solely utilizing motif matching or sequence-based machine learning models, which prevents them from learning the whole complex features of degrons, especially the structural properties. In addition, none of the previous methods build an online responsive platform to provide timely and customized predictions for E3 targeted degron, restricting its capability for degron identifications. In this study, we present the MetaDegron, a novel bioinformatics tool accompanied by a user-friendly web service, designed to predict E3 targeted degron. MetaDegron incorporates comprehensive featurization strategies and leverages the protein language model to identify novel degron instances, which was trained on a curated dataset of 300 degron instances. Moreover, extensive evaluation and comparative analysis demonstrate the superior performance of MetaDegron. Functionally, MetaDegron offers the convenience of batch prediction for targeted degrons associated with 21 E3 ligases. Furthermore, the web service provides functional annotations and visualization tools for a range of degron-related structural and physicochemical features. MetaDegron can be accessed freely at http://modinfor.com/MetaDegron/ and https://github.com/BioDataStudy/MetaDegron, enabling broad accessibility to the research community and facilitating exploration within the fields of biological mechanisms, protein degradation implications, and degron-centric drug development.

## Methods

### Data preparation

We collected and processed a set of human degron motifs, which are E3 binding consensus patterns, from the ELM database [36], and over 300 degron instances from a number of previous studies (Supplementary Table S2) [14, 30, 32, 37]. For instance, β-TrCP2 (E3, known as FBXW11) recognizes DSGxxS consensus degron motif, where x denotes any one of the 20 amino acids. In order to improve the characterization of degrons, we constructed a comprehensive background dataset comprising a considerable number of randomly selected peptides with the same length as the degron instances (Supplementary Table S3). This strategy enabled us to perform comparative analysis based on peptide sequences of similar length, thus providing a suitable control to evaluate the specificity of degron sequences. To further investigate the structural and physicochemical properties associated with the curated degrons, multiple analyses were performed [38–45]. We calculated 10 features for all motif instances or random peptides. Specifically, the determination of residue-specific flexibility utilized the DynaMine software [40] employing default parameters. Residue-specific solvent accessibility and secondary structures, including coiled coil and α-helix, were computed using the Spider2 tool [39]. Protein disorder was assessed through the utilization of the IUPred software [38]. The anchoring score of each degron was evaluated employing the ANCHOR program [46]. Multiple sequence alignments (MSA) of orthologous proteins were acquired utilizing the Gopher tool from Bioware [47] to calculate sequence conservation. Information pertaining to protein domains was retrieved from the Pfam database [41]. Moreover, we evaluated the enrichment of important post-translational modifications (PTMs, phosphorylation and ubiquitination) within and around degrons. The experimentally verified PTMs information was downloaded from our constructed Eukaryotic Phosphorylation Sites Database [43] and Protein Lysine Modifications Database resources [42], respectively. By comprehensively analysing and elucidating the properties of these degrons, we can gain valuable insights into their sequence motifs, structural features, and biochemical properties. Such insights not only enhance our understanding of protein degradation but also facilitate the development of more accurate prediction models. To facilitate the development and evaluation of our models, the constructed dataset was partitioned into a training dataset, which represented 90% of the total data, and an independent dataset, which accounted for the remaining 10% of the data.

### Model architecture

Based on curated degrons and random peptides, we employed a bootstrapping strategy to train 10 eXtreme Gradient Boosting (XGBoost) classifiers [48], leveraging multiple distinguishing features between these two groups. Exhaustively exploring numerous parameter combinations for each classifier, we then selected the optimal parameters through multiple rounds of cross-validation (CV) assessments. The average prediction scores of these 10 models were employed to establish the ultimate probability of degron occurrence. To ensure that the model can make predictions when the protein features are not matched, we extended its functionality by incorporating a deep neural network (DNN).

The DNN architecture employed by MetaDegron comprises two distinct components, as illustrated in Fig. 3. The initial component utilizes context-sensitive embeddings of amino acids generated by Embeddings from Language Models (ELMo) [49], known for its efficacy in various protein sequence prediction tasks. ELMo is a contextual word embedding technique originally developed for natural language processing tasks. Here, we used SeqVec, a protein-specific adaptation of ELMo, leverages bidirectional Long Short-Term Memory (BLSTM) networks to generate context-sensitive embeddings for amino acid sequences. By modeling protein sequences, SeqVec effectively captured the biophysical properties of the language of life from unlabeled big data (UniRef50), which allows SeqVec to generate embeddings that encode the contextual dependencies between amino acids across the entire sequence. Each amino acid in a given protein sequence is encoded into a 1024-dimensional vector, encapsulating chemical, physical, and structural information. These embeddings are then used as inputs for a BLSTM layer and a convolution-pooling layer, which are sequentially connected to a dense layer for feature integration.

By employing convolutional layers followed by max-pooling operations, the convolutional neural network (CNN) component of our model can effectively extract hierarchical features representing short-range interactions between amino acid residues. These features capture local sequence motifs that may be indicative of E3 ligase targeting signals or degron recognition motifs. The second component of the DNN architecture involves encoding each amino acid into an adjustable-length vector, which is context-insensitive and undergoes joint training with the rest of the DNN. This embedding layer is connected to a BLSTM layer followed by another dense layer. Here, we leverage word embedding techniques, inspired by natural language processing, to transform each amino acid residue within a peptide segment into a dense, continuous vector representation. Similarly, in the context of peptide sequences, we can exploit the inherent similarities and relationships between amino acids to learn meaningful representations that capture their contextual dependencies. By integrating a BLSTM network with the word embedding model, we enable the model to capture both forward and backward sequential dependencies within peptide sequences. Subsequently, the two dense layers are further connected to an additional pair of dense layers, ultimately culminating in an output layer with two nodes representing the degron and random peptide classes.

### Model evaluation

In this study, we adopt a rigorous validation framework that encompasses both CV techniques and independent test sets to comprehensively evaluate the performance of MetaDegron. Firstly, we employed five-fold CV, a widely used technique in machine learning, to assess the model’s performance on the training dataset. This involves partitioning the dataset into k subsets, training the model on k-1 subsets, and evaluating its performance on the remaining subset. This process is repeated k times, with each subset serving as the validation set once. By averaging the performance metrics across multiple iterations, we obtain a more reliable estimate of the model’s predictive performance and robustness to variations in the training data. Specifically, For the MetaDegron model, receiver operating characteristic (ROC) curves were constructed for each subset and five ROC curves were generated, and subsequently, a mean ROC curve was calculated, ensuring that each of the five models carried equal weight. In order to assess the model’s generalizability, ROC curves were plotted, and the corresponding area under the ROC curve (AUC) values were determined using an independent dataset. In general, the AUC values range from 0 to 1, while a higher AUC value indicates a higher accuracy of a predictive model. Moreover, additional metrics, e.g., the area under the precision-recall curve (AUPRC), accuracy, recall and F1-score, were also calculated for a comprehensive comparative analysis. We also compared the performance of the MetaDegron with previous method using an additional dataset comprising experimentally validated substrates of β-TrCP2 (Supplementary Table S4). To avoid the possible bias caused by a random sampling from the background dataset, the random sampling was performed 10 times, and the same number of random peptides with the same length was extracted each time. We calculated the average AUC, AUPRC, accuracy, recall, and F1-score.

### Website implementation

The MetaDegron web server was developed following the standard Model-View-Controller framework, a prevalent approach in contemporary web application design [50]. This framework was structured into three primary logical components, namely ‘Prediction’, ‘Results’, and ‘Controller’, which collectively constitute the MetaDegron system. On the backend, the system integrates two well-optimized models, specifically XGBoost and deep learning models denoted as MetaDegron-X and MetaDegron-D, tailored for the prediction of E3 ligase-targeted degrons. The ‘Controller’ module serves a pivotal role by validating the input data’s format, facilitating the transfer of data from the frontend interfaces to the backend, orchestrating the execution of predictive models, and ultimately delivering the results to the ‘Results’ page. The ‘Prediction’ component, positioned as the frontend interface, enables user interactions with the system. To ensure a responsive server, both the ‘Prediction’ and ‘Results’ interfaces were constructed using an amalgamation of HTML5, CSS (utilizing Bootstrap3), and JavaScript. jQuery, a JavaScript library, was employed to leverage Ajax technology for seamless communication with the ‘Controller’ module. Additionally, PHP was employed as a complementary tool for the presentation of results. In the ‘Results’ page, the MetaDegron provides the functional annotations and visualizations of degron-related features and source protein. For the degron, the properties calculated by MetaDegron are provided for users. For the source protein, the base information is obtained to display from the UniProt database. The interacting proteins of source protein, including E3s and deubiquitinating enzymes (DUBs) are provided with a tabular list and an interactive network based on the Cytoscape.js [51]. For both degron and source protein, the structural and MSA information are visualized by 3Dmol.js [52] and ProViz tool [53], respectively.

## Results

### Overview of the MetaDegron framework

**Figure 1:**
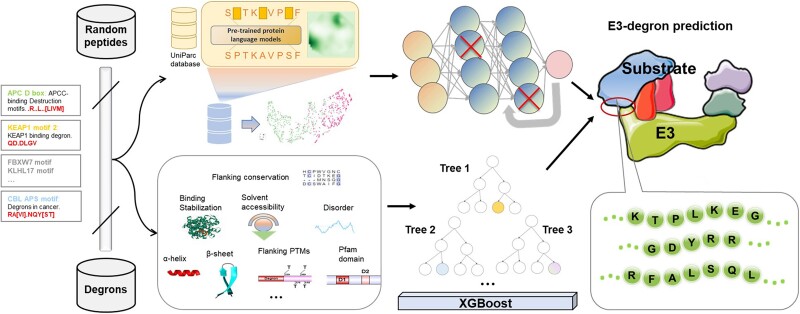
The overall framework of MetaDegron.

MetaDegron is a novel tool specifically designed for precise degron prediction using machine learning techniques (Fig. 1). It comprises two distinct models: MetaDegron-D and MetaDegron-X, each employing different methodologies for degron prediction. MetaDegron-D operates by extracting features solely from the protein sequence. The input to this model is peptide segments that are centered around the degron region. We employ a pre-trained protein model to represent each degron, which generates context-sensitive embeddings in a 1024-dimensional space. These embeddings undergo further processing using various feature extraction networks such as convolution-pooling or BLSTM layers. In addition, another word embedding model integrating BLSTM network was utilized to convert the peptide segment into a 27-dimensional vector for pattern extraction. These two components are combined through pairing of these fully connected networks, resulting in the final degron prediction. On the other hand, MetaDegron-X takes into consideration multiple distinctive features of a degron itself. This model employs input features that encompass various aspects of the degron, including sequence, evolution, and structure. To achieve this, an XGBoost classifier is employed for final prediction. Overall, MetaDegron possesses the capability to predict targeted degrons of 21 E3 ligases in a batch manner, and provides functional annotations and visualization of multiple degron-related structural and physicochemical features. The comprehensive functionality of MetaDegron allows researchers to gain crucial insights into the functional aspects of degrons and their relation to a wide range of protein characteristics.

### Characterization and prediction of degron with structure characteristics

**Figure 2:**
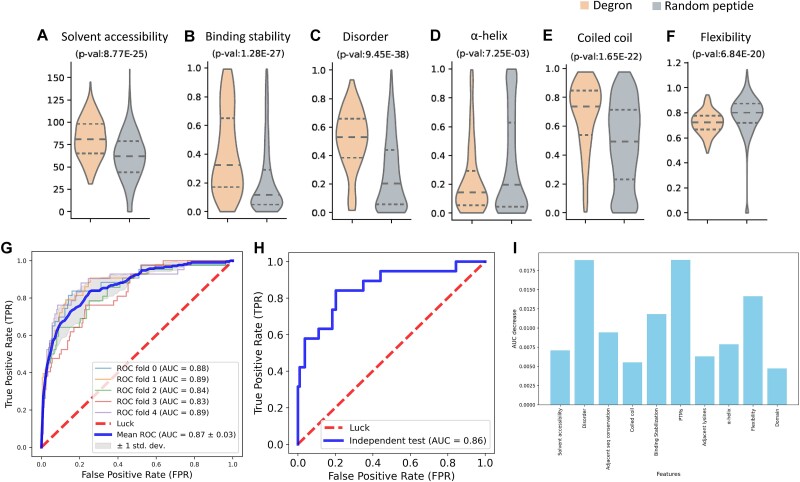
Performance of MetaDegron based on protein features. (A-F) the statistics of multiple characteristic features for the known degron instances and random peptides, including solvent accessibility (A), binding stability (B), disorder (C), α-helix (D), coiled coil (E), and flexibility (F). (G-H) ROC curves for MetaDegron-X in different five-fold CV (G) and independent testing dataset (H). (I) Elimination study to assess the association between each feature and the prediction results.

Targeted degrons play pivotal roles in regulating protein stability and turnover within the cell, influencing various cellular processes. Understanding the structure characteristics of degrons is crucial for predicting their degradation potential and unraveling their functional implications. To characterize the structure features of degrons, we analyzed curated, experimentally validated degrons by comparing them to a background dataset. We employed various structural bioinformatics algorithms and tools to identify common structural properties within degrons [36, 38, 39, 41–43, 45]. Remarkably, the known degrons exhibited a higher degree of solvent accessibility and binding stability compared to the random peptides (Fig. 2A and B), suggesting their importance in recognition by degradative enzymes. Furthermore, degrons were found to be preferentially located in protein disordered regions (Fig. 2C), highlighting their distinctive localization patterns. Additionally, the analysis revealed a specific preference of degrons for coiled coil regions rather than α-helix regions (Fig. 2D and E). It was also observed that degrons tend to occur in lower flexibility regions (Fig. 2F). These findings provided valuable insights into the structural characteristics of degrons and indicate potential determinants for degron recognition and degradation. Subsequently, the XGBoost classifier (called MetaDegron-X) was constructed using these discerning features for E3 targeted degron. The performance of MetaDegron-X, as assessed by the AUC values, was promising. Specifically, the AUC values ranged from 0.83 to 0.89 in a five-fold CV, with an average AUC value of 0.87 (Fig. 2G). Furthermore, validation of the developed MetaDegron-X was carried out on an independent testing dataset. The performance of MetaDegron-X was superior, as denoted by the AUC value of 0.86 (Fig. 2H). These findings collectively demonstrated the high accuracy and robust performance achieved by MetaDegron-X.

In addition, we conducted an elimination study to assess the association between each feature and the prediction results. Specifically, we systematically removed one feature at a time from the input data and evaluated the impact on the predictive performance of our model. By quantifying the change in AUC value through five-fold CV, we revealed the relative importance of each feature in predicting E3 ligase targeted degrons (Fig. 2I). From the results, we found all features contribute to the construction of the model, i.e. the performance of the models decreased to different degrees after removing the specific features. Overall, those features, such as disorder and the number of PTMs within the degron region, represented the most important features. This is consistent with the facts that degrons are preferentially located in disordered regions and regulated by PTMs that control the interaction with E3s in response to environmental and cellular cues.

### Enhancing the MetaDegron system using deep learning technology

**Figure 3:**
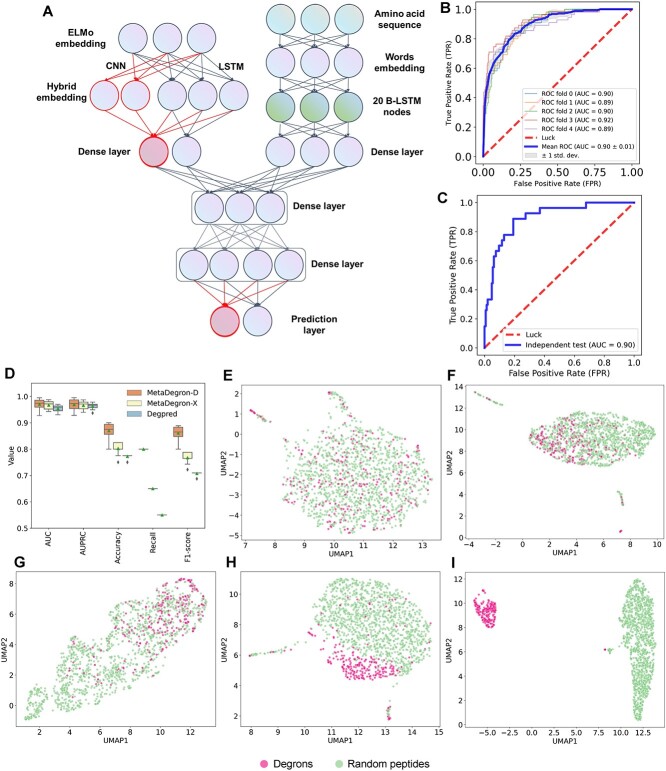
Implementation and performance of MetaDegron based on deep learning framework. (A) The hybrid deep learning architecture of MetaDegron. (B-C) ROC curves for MetaDegron-D in different five-fold CV (B) and independent testing dataset (C). (D) Comparison of MetaDegron with Degpred on the additional independent dataset. (E-I) feature representation of the known degrons and random peptides using the UMAP method in each network layer, including input layer (E), dense layer (context-sensitive) (F), dense layer (context-insensitive) (G), joint features layer (H), dense layer (before output) (I).

The inability to match protein features on certain occasions may pose a challenge for feature-driven MetaDegron-X to achieve prediction. To address this issue and enhance the prediction capabilities of the model, we have developed an extended version called MetaDegron-D. By incorporating a deep learning framework (see ‘Methods’ part), MetaDegron-D was capable of solely operating on protein sequences. This novel approach utilized a hybrid architecture comprising cutting-edge deep learning networks (Fig. 3A), such as protein language models, word embeddings, convolution, and BLSTM, as thoroughly detailed in the methodology section. This deep learning framework allows MetaDegron-D to leverage the full potential of these advanced networks and their ability to extract high-level features from protein sequences. The performance evaluation of MetaDegron-D demonstrated its great predictive capabilities. Through a five-fold CV approach, we obtained an average AUC value of 0.90. Furthermore, the AUC values ranged from 0.89 to 0.92, indicating consistent and reliable performance (Fig. 3B). Additionally, when tested with an independent dataset, MetaDegron-D achieved an improved AUC value of 0.90 (Fig. 3C). These results suggested the robustness and accuracy of MetaDegron-D in predicting protein features solely from the sequence information.

Moreover, we assessed the performance of the MetaDegron using an additional dataset comprising experimentally validated substrates of β-TrCP2, as detailed in Supplementary Table S4. We first extracted all reported substrates, and matched and filtered those with an instance of β-TrCP2 degron within their protein sequence. A subset consisting of 20 substrates exhibiting the presence of the β-TrCP2 degron was designated as the positive dataset. We also constructed a background dataset comprising over 700 randomly selected peptides with the same length as the degron instances (Supplementary Table S4). Using this new benchmark dataset, we compared MetaDegron to Degpred and found that MetaDegron could achieve better AUROC value (Fig. 3D). The AUC values were computed as 0.9705 (ranged from 0.9275 to 0.9950, MetaDegron-D), 0.9670 (ranged from 0.9425 to 0.9875, MetaDegron-X), and 0.9540 (ranged from 0.9300 to 0.9700, Degpred), respectively. We also computed the additional metrics, e.g. AUPRC, accuracy, recall, and F1-score, for our models and Degpred. MetaDegron still outperformed Degpred in term of these metrics (Supplementary Table S5). Through extensive evaluation on benchmark datasets and comparison with Degpred, we demonstrated the superior performance of MetaDegron.

To further explore the capabilities of the MetaDegron framework, we utilized the visualization method described by Becht et al. [54] to compare the features of degrons and random peptides across each network layer (Fig. 3E-I). As expected, the feature representations of the input layer for both degrons and random peptides exhibited significant overlap and mixing (Fig. 3E). However, as the framework underwent training, a clear distinction between degrons and random peptides emerged, resulting in more separated clusters within the feature space (Fig. 3I). This observation emphasizes the effectiveness of the MetaDegron-D model in identifying efficient features and differentiating between degrons and random peptides.

### The usage of MetaDegron

**Figure 4:**
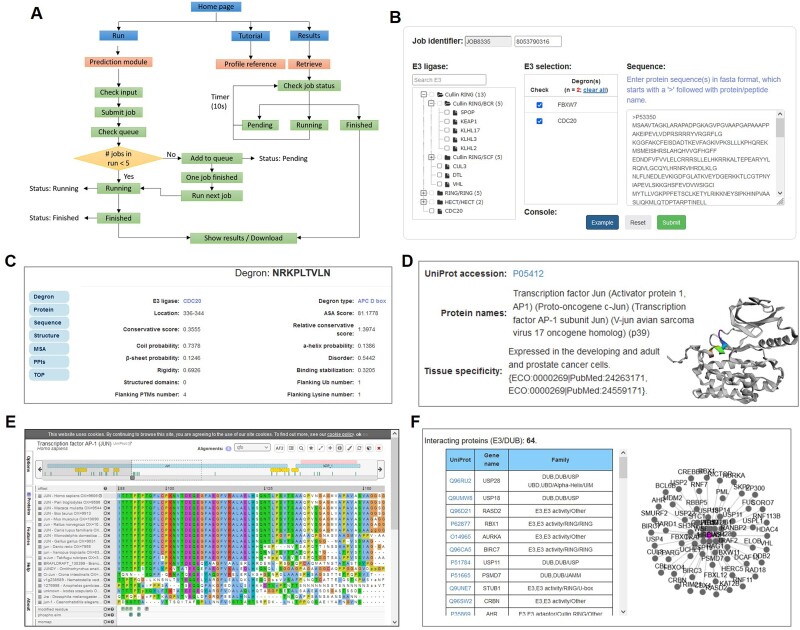
The usage of MetaDegron webserver. The general pipeline for MetaDegron server. (B) The example selection of E3s and sequences in the ‘prediction’ page. (C) The feature properties of selected degron. (D-F) The annotations of source protein for selected degron, including the base and structure information (D), the MSA viewer (E), and the interacting annotations (F).

MetaDegron serves as a useful tool for predicting targeted degrons of 21 E3 ligases, offering researchers possible candidates for studying protein degradation pathways and identifying potential therapeutic targets. The multimodal feature integration approach enables MetaDegron to capture diverse aspects of degron recognition, including amino acid composition, physicochemical properties, evolutionary conservation, and contextual dependencies, thereby enhancing its capabilities for advancing research in the field of protein degradation and ubiquitin-mediated proteolysis. The webserver of MetaDegron was designed and constructed with a modular and user-friendly manner (Fig. 4). Three major modules, including ‘Run’, ‘Results’ and ‘Tutorial’, are the kernel of MetaDegron online server (Fig. 4A). The ‘Run’ module sequentially controls the execution of submitted jobs, including the input checking, job submitting, job running, and task terminates. Meanwhile, the ‘Results’ module records the submission jobs, monitors the status of jobs, and immediately shows the prediction results. The clickable and searchable hierarchical classification tree of E3s is loaded for the selection of single or multiple E3 ligases (Fig. 4B). Then, one or more protein sequences in FASTA format can be submitted. After finishing the submitted job, the prediction results will be visualized with specific information, including the ‘Entry’, ‘E3 ligase’, ‘Degron instance’, ‘Degron type’, ‘Start’, ‘End’, and ‘Score’. It displays the detailed information for degron and source protein (Fig. 4C-F). The properties of degron (Fig. 4C) and information of source protein (Fig. 4D) are displayed as well. In addition, the structure of source protein is presented with 3Dmol.js [52], and the degron instance is marked with highlights. Moreover, the MSA of degron instance and source protein are visualized by using the ProViz tool [53] (Fig. 4E), and the interacting E3s or DUBs of source protein are provided in a tabular list and an interactive network based on the Cytoscape.js [51] (Fig. 4F). Taken together, MetaDegron is a user-friendly online tool for the study of targeted protein degradation.

## Discussion

Targeted protein degradation represents a highly promising therapeutic modality that is currently gaining considerable attention in the biomedical fields [5, 8, 55–60]. In the ubiquitin proteasome system, the interactions between E3 ligase and the degradation signal (degron) is critical for determining degradation specificity and maintaining cellular homeostasis. In this study, we develop a new approach, called MetaDegron, for predicting E3 targeted degron, based on our careful data collection, curation, and analysis. The built-in models of MetaDegron integrate comprehensive featurization strategies and large protein language model, displaying great performance in both machine learning and deep leaning models. MetaDegron represents a novel framework that integrates state-of-the-art protein language models with a diverse array of featurization techniques to enhance the predictive accuracy of E3 ligase targeted degrons. By leveraging protein language models, MetaDegron can effectively extract high-dimensional embeddings that encode rich contextual information from the input protein sequences. Moreover, MetaDegron utilizes a multimodal feature integration approach, which combines contextual information, sequence-based features, and structural features to improve prediction performance. This innovative tool first time enables users to perform batch prediction of targeted degrons for 21 E3 ligases, while it also provides functional annotations and visualizations of various degron-related structural and physicochemical features.

Our structural analyses in this study help a deep understanding of the structural characteristics of degrons. Accordingly, we developed a robust computational method, MetaDegron-X, for predicting E3-targeted degrons. The identification of degron structural properties and the development of accurate prediction tools are crucial steps towards unraveling the regulatory mechanisms underlying protein degradation processes and may have implications for drug discovery and biomedical research [15, 61]. Moreover, our extended MetaDegron-D model demonstrates excellent predictive performance, indicating its potential to overcome the challenges posed by mismatched protein features. The incorporation of a deep learning framework enables MetaDegron-D to operate solely on protein sequence information and achieve accurate predictions. The visualization of degrons and random peptides further supports the efficacy of the MetaDegron-D model in distinguishing between these two classes based on their specific features.

The protein homeostasis primarily depends on the protein degradation via UPS, and the aberrant regulations of protein homeostasis can lead to various diseases, including cancer, neurodegenerative disorders, and inflammatory conditions. Predicting E3 ligase targeted degrons holds significant promise for accelerating our understanding of protein degradation pathways and facilitating the discovery of novel therapeutic targets. By identifying specific sequences recognized by E3 ligases for ubiquitination and subsequent degradation, MetaDegron enables the characterization of substrate specificity and regulatory mechanisms of E3 ligases, shedding light on their roles in protein degradation and homeostasis. In addition, predicting E3 ligase targeted degrons can provide critical insights into the aberrant protein turnover mechanisms driving oncogenesis and tumor progression. By elucidating the substrate specificity and regulatory networks of E3 ligases in cancer cells, researchers can identify therapeutic targets for precision oncology interventions. Moreover, MetaDegron’s predictive capabilities have implications for drug discovery and development efforts targeting protein degradation pathways. By enabling the identification of candidate substrates for E3 ligases, MetaDegron can facilitate the screening and prioritization of compounds that modulate protein degradation processes. Finally, MetaDegron may aid in the design of targeted therapies aimed at selectively inducing protein degradation of disease-associated proteins, offering a new paradigm for drug development. Overall, predicting E3 ligase targeted degrons has far-reaching implications for basic research, translational studies, cancer research, and drug development. By providing insights into degradation mechanisms, identifying therapeutic targets, and guiding precision medicine approaches, we anticipate that the MetaDegron can serve as a useful tool to identify E3-targeted degrons for further research of protein regulation and drug development.

There are some limitations in this study. First, degrons are short linear motifs specifically recognized by E3s; therefore, the proteins with similar degrons may be recognized by a specific E3 to further off-target degradation. However, the interaction of E3 and degron really provides the potential approach to degrade the drug target. Meanwhile, the off-target effects of the predicted molecule can be reduced in the design of targeted drug, such as the consideration of more molecular features of target protein, the discovery of ligand, and the development the molecular glue. Another potential limitation lies in the biases present in the datasets used for training and evaluation. Experimental studies characterizing degron sequences may exhibit biases towards certain protein families, cellular contexts, or experimental techniques. Consequently, the predictive performance of our model may be influenced by the distribution of data across different classes and may not fully generalize to unseen data or underrepresented classes. Especially, the number of known degron instances, reported E3-degron interactions, and E3-substrate interactions are still limited. In addition, our approach relies on the integration of diverse features, including sequence-based, structural, and contextual features, to characterize degron sequences comprehensively. However, the selection and representation of these features may introduce implicit assumptions about the underlying mechanisms of E3 ligase targeting and degron recognition. While we aim to capture a broad range of sequence-structure relationships, our feature representation may not fully capture all relevant aspects of degron recognition, leading to potential limitations in predictive performance. Also, while our model provides accurate predictions of E3 ligase targeted degrons, the interpretability of these predictions may be limited. It may be challenging to elucidate the underlying biological mechanisms, especially for complex or novel sequences. More experimental validation is necessary to further optimize the reliability of the predictions. Enhancing the interpretability of our model’s predictions could improve its utility for guiding experimental validation and hypothesis generation. In terms of performance comparison, both deepDegron and DEGRONOPEDIA were developed for predicting N-/C-degrons, whereas only Degpred supported the prediction of internal degron and E3-degron interactions. Thus, only Degpred tool was used for performance comparison in this study. We expect more tools to be developed in the future, leading to more extensive comparisons.

In future, more useful features and machine learning frameworks will be adopted for the improvement of MetaDegron models. We expect more E3 and their targeted degrons will be discovered, and they will be integrated into our framework to extend the benchmark dataset and improve the performance of MetaDegron. Moreover, the functional prediction of E3s and targeted degrons should be considered by combining the high-throughput omics and computational prediction. We will continuously maintain and improve the server of MetaDegron.

**Key Points**

- We integrate the protein language model and comprehensive featurization strategies to develop a novel framework, namely MetaDegron for the identification of new degron for targeted protein degradation.
- MetaDegron covers 21 E3 ligases-targeted degron predictions, showing excellent performance through extensive evaluation and comparative analysis.
- MetaDegron implements an online prediction website and provides functional annotations and visualization of multiple degron-related structural and physicochemical features.

## Supplementary Material

Supplementary_Table_S1_bbae519

Supplementary_Table_S2_bbae519

Supplementary_Table_S3_bbae519

Supplementary_Table_S4_bbae519

Supplementary_Table_S5_bbae519

## Data Availability

MetaDegron can be accessed freely at http://modinfor.com/MetaDegron/ and https://github.com/BioDataStudy/MetaDegron.
